# Ocular surface manifestation of COVID-19 and tear film analysis

**DOI:** 10.1038/s41598-020-77194-9

**Published:** 2020-11-19

**Authors:** Alessandro Meduri, Giovanni William Oliverio, Giuseppe Mancuso, Angela Giuffrida, Claudio Guarneri, Emmanuele Venanzi Rullo, Giuseppe Nunnari, Pasquale Aragona

**Affiliations:** 1 Biomedical Science Department, Institute of Ophthalmology, University of Messina, Via Consolare Valeria, 1, Messina, Italy; 2 Department of Human Pathology in Adulthood and Childhood “Gaetano Barresi” Division of Microbiology, University of Messina, Messina, Italy; 3 Department of Clinical and Experimental Medicine, Unit of Infectious Diseases, A.O.U. ”G. Martino”, University of Messina, Messina, Italy

**Keywords:** Conjunctival diseases, Corneal diseases

## Abstract

To evaluate the ocular manifestation in patients hospitalized with coronavirus disease 2019 (COVID-19) and to search for the presence of severe acute respiratory syndrome coronavirus 2 (SARS-CoV-2) in tears. This study was conducted in 29 hospitalized patients who were admitted to the COVID center at the Policlinic Hospital of the University of Messina, Italy. All patients underwent an ophthalmologic assessment comprising a Standardized Patient Evaluation of Eye Dryness (SPEED) questionnaire, anterior segment, and the ocular surface examination of both eyes using a portable slit lamp. The Schirmer I test was performed, and the filter paper strip was used to search for the presence of SARS-CoV-2 on the ocular surface by real-time quantitative polymerase chain reaction (RT-qPCR). A total of 10 patients reported ocular symptoms; in particular, four reported eye burning, three reported foreign body sensation, and three reported tearing. Moreover, seven patients presented conjunctival hyperemia and/or chemosis, eleven patients presented blepharitis signs such as lid margin hyperemia and/or telangiectasia, crusted eyelashes, and meibomian orifices alterations. Tear analysis did not reveal the presence of SARS-CoV-2. Ocular symptoms are common in patients with COVID-19; although, tear analysis did not reveal the presence of SARS-CoV-2.

## Introduction

The novel coronavirus, named severe acute respiratory syndrome coronavirus 2 (SARS-CoV-2), was identified for the first time in December 2019 after a series of acute atypical pneumonia cases occurred in Wuhan, China^1^. Following this outbreak, SARS-CoV-2 quickly spread worldwide, became a public health emergency, and a pandemic was declared by the World Health Organization (WHO). SARS-CoV-2 presents characteristics similar to those of severe acute respiratory syndrome coronavirus (SARS-CoV), which was responsible for the SARS outbreak that occurred in 2002–2003^2^.

Human angiotensin-converting enzyme 2 (ACE2) has been recognized as a functional receptor, and similar to SARS-CoV, SARS-CoV-2 is capable of binding and using ACE2 as a cell entry receptor to epithelial cells via the spike (S) protein^3^. Since a high expression level of ACE2 has been demonstrated in respiratory and alveolar epithelial cells, oral mucosa, gastrointestinal duct, kidney, and conjunctiva, these represent potential infection sites of SARS-CoV-2^4^. Furthermore, the main route of transmission is respiratory droplets and direct contact.

Recent studies have demonstrated detection of the virus in tears and conjunctival secretions, leading to the suggestion that ocular exposure may be a potential route of infection via the nasolacrimal duct to the respiratory tract^5,6^.

The clinical features of patients with a confirmed diagnosis of coronavirus disease 2019 (COVID-19) range from minimal symptoms to severe respiratory and multi-organ failure^7^. The most commonly reported symptoms are fever, cough, shortness of breath, and dyspnea; however, several less common non-respiratory clinical features have also been described, such as diarrhea and vomiting, and more rarely, conjunctivitis^7^. Recently numerous reports in the literature documented the ocular surface and eye involvement in patients with COVID-19^8–15^.

In the present study, we report the ocular symptoms and findings in 29 patients with confirmed COVID-19 and search for the presence of SARS-CoV-2 in tears by real-time quantitative polymerase chain reaction (RT-qPCR).

## Results

A total of 29 patients were evaluated (14 females, and 15 males). The mean age of the patients was 77.1 ± 12.6 years (range 44 − 92 years). Seven patients presented with severe COVID-19 disease, while 22 had moderate disease. The mean duration of disease was 17.4 ± 12.1 days (Table 1).

**Table 1:** Clinical characteristics of the study population.

Age, years	77.1 ± 12.6
Male, *n* (%)	15 (51.7%)
Disease severity	
Severe, *n* (%)	7 (24.1%)
Moderate, *n* (%)	22 (75.9%)
Duration of COVID-19, days	18.7 ± 7.9
0 − 10 days	5 (17.2%)
10 − 20 days	11 (38%)
> 20 days	13 (44.8%)
Temperature, °C	37.3 ± 0.8
Comorbidities	
Hypertension, *n* (%)	25 (86.2%)
Diabetes, *n* (%)	18 (62.1%)
Obesity, *n* (%)	13 (44.8%)
Coronary heart disease, *n* (%)	8 (27.5%)
Cerebrovascular disease, *n* (%)	7 (24.1%)
Atrial fibrillation, *n* (%)	7 (24.1%)
Neurological disease, *n* (%)	4 (13.8%)
Cancer, *n* (%)	9 (31%)
BPCO, *n* (%)	9 (31%)
Smoker, *n* (%)	5 (17.2%)
Chronic renal disease, n (%)	7 (24.1%)
Therapy	
Antibiotics, *n* (%)	26 (89.6%)
Antiviral, *n* (%)	9 (31%)
Corticosteroids, *n* (%)	13 (44.8%)
Low molecular weight heparin, *n* (%)	26 (89.6%)
Hydroxychloroquine, *n* (%)	19 (65.5%)

n = number of patients; temperature refers to the measurement during ophthalmologic evaluation.

### Results of the ophthalmologic assessment

The mean value for the Schirmer I test was 9.2 ± 6.4 mm/5 min in the right eye and 9.3 ± 6.4 mm/5 min in the left eye. The mean Standardized Patient Evaluation of Eye Dryness (SPEED) questionnaire (composite) score was 2.3 ± 1.7. Ten patients described ocular symptoms following the onset of COVID-19; in particular, four reported eye burning, three reported foreign body sensation, and three reported tearing. In 17 patients, ocular findings were observed, such as conjunctival hyperemia, ocular secretion, lid margin hyperemia and/or telangiectasia, crusted eyelashes, and meibomian orifices alterations; however, only five patients who reported ocular symptoms actually presented with evident ocular manifestations at the ophthalmologic evaluation.

Seven patients presented conjunctival hyperemia and or chemosis, eleven patients presented blepharitis signs.

Only one patient presented with conjunctivitis with moderate hyperemia, chemosis, and secretions (Table 2). No patients reported a loss of vision. Three patients reported a history of diabetic retinopathy, and one patient had glaucoma that was being treated with latanoprost eye drops in both eyes. Seven patients had previously undergone cataract surgery, all of whom reported ocular symptoms. No significant difference was observed in patients with and without previous eye disease regarding ocular symptom assessment (*p* = 0.98) (Table 3).

**Table 2:** Ophthalmologic assessment.

Distance spectacles, *n* (%)	10 (34.5%)
Previous ocular surgery, *n* (%)	8 (27.6%)
Previous ocular disease, *n* (%)	3 (10.3%)
Patients with ocular symptoms, *n* (%)	10 (34.5%)
SPEED questionnaire	
Severity	1.21 ± 0.9
Frequency	1.14 ± 0.8
Total	2.35 ± 1.7
Ocular symptoms	
Foreign body sensation, *n* (%)	3 (10.3%)
Ocular pain, *n* (%)	3 (10.3%)
Dryness, *n* (%)	2 (6.9%)
Tearing, *n* (%)	5 (17.2%)
Ocular findings	
Hyperemia, *n* (%)	7 (24.1%)
Chemosis, *n* (%)	1 (3.4%)
Secretion, *n* (%)	2 (6.9%)
Lid margin hyperemia, *n* (%)	10 (34.5%)
Crusted eyelashes, *n* (%)	7 (24.1%)
Meibomian orifices abnormalities, *n* (%)	6 (20.7%)
Schirmer I test, mm/5 min	
Right eye	9.2 ± 6.4
Left eye	9.3 ± 6.4

n = number of patients; SPEED questionnaire = Standardized Patient Evaluation of Eye Dryness; mm = millimeters; hyperemia, chemosis, and secretion refer to conjunctival findings.

**Table 3:** Comparison of ocular findings and risk factors.

	Ocular symptom/ocular manifestations		*p* value
	Yes	No	
Distance spectacles			**.002**
Yes	4	6	
No	18	1	
Previous ocular surgery			.14
Yes	7	0	
No	15	7	
Previous ocular disease			.98
Yes	2	1	
No	20	6	
Previous eye drop use			.24
Yes	0	1	
No	22	6	
COVID-19			.14
Severe	7	0	
Moderate	15	7	

*P*-value was evaluated using Fisher’s Exact test. Significant data are reported in bold.

### Tear sample analysis results

In all evaluated tear samples, the control sample presented a C(t) between 24 and 27; however, no analyzed tear sample showed a positive result for SARS-CoV-2. The nasopharyngeal swab, evaluated on the same day as tear collection, was negative in four patients and positive in 25 patients.

### Correlation between ophthalmologic findings and COVID-19

A significant correlation was found between conjunctival hyperemia and severity of the disease (ρ_s_ = 0.55; *p* = 0.002), and between blepharitis-like findings and duration of the disease (ρ_s_ = -0.54; *p* = 0.003). The composite SPEED score was significantly correlated with the presence of blepharitis findings (ρ_s_ = 0.95 *p*; < 0.0001), but not with conjunctival findings (*p* = 0.11) (Table 4).

**Table 4:** Correlation between ocular findings and duration and severity of disease.

	Duration		Severity		SPEED questionnaire (composite)	
	ρ_s_	*p* value*	ρ_s_	*p* value*	ρ_s_	*p* value*
Conjunctival signs ^a^	0.01	0.96	0.55	**.002**	0.21	.11
Blepharitis-like findings ^b^	− 0.54	**.003**	− 0.005	.99	0.95	** < .0001**
Schirmer I ^c^	0.18	.36	− 0.25	.21	− 0.52	.12
SPEED questionnaire						
Frequency	− 0.28	.26	0.29	.30		
Intensity	− 0.30	.23	0.26	.36		
Composite	0.46	.16	0.31	.37		

^a^Conjunctival hyperemia and chemosis; conjunctival chemosis; ^b^Ocular secretion; lid margin hyperemia and/or telangiectasia; crusted eyelashes, meibomian orifices abnormalities; ^c^Values from the right eye.

*Spearman rank correlation analysis; Significant data are reported in bold.

## Discussion

Numerous respiratory viruses, such as adenovirus, influenza virus, respiratory syncytial virus, coronavirus, and rhinovirus, exhibit an ocular tropism, which could be responsible for ocular infections^16^. Although previous studies have documented the presence of coronavirus in tears from patients with SARS-CoV infection, specific ocular involvement has not been described with either Middle-East respiratory syndrome coronavirus (MERS-CoV) or SARS-CoV^17–20^.

Similar to other coronaviruses, the main route of transmission of SARS-CoV-2 is respiratory droplets and direct contact; however, the human ocular surface may represent a further exposure site. In fact, infectious droplets and bodily fluids can easily contaminate the corneal − conjunctival epithelium^21^. Furthermore, ocular tissue could be a potential site of viral replication due to the expression of ACE-2 receptors, which can supposedly bind SARS-CoV-2 to cause infection^22^.

The ocular surface is able to create a defensive barrier against several pathogens via the production of antimicrobial peptides such as immunoglobulins, lysozyme, and lactoferrin, as well as the presence of normal components of the bacterial flora^23–25^. Moreover, the mucin layer helps to avoid the binding of the virus to its epithelial receptors, potentially promoting its clearance through nasolacrimal drainage and acting as a gateway for the transfer of SARS-CoV-2 to the nose and respiratory mucosa^23,24^.

In the present study, we document a high prevalence of mild ocular symptoms reported by patients diagnosed with COVID-19. Furthermore, ocular assessment demonstrated the presence of mild conjunctival hyperemia, chemosis, and secretions, as well as the occurrence of eyelid margin hyperemia and other signs referable to blepharitis. On the other hand, ocular findings such as eyelid margin hyperemia and crusted eyelashes, as well as symptoms such as ocular dryness, burning, and foreign body sensation are common in the general population, especially considering the mean age of our cohort, suggesting a possible overlap with ocular surface diseases such as dry eye and meibomian gland dysfunction. However, all patients confirmed a worsening in ocular symptomatology following onset of the disease. In fact, a significant correlation between the presence of blepharitis findings and SPEED questionnaire score was observed (*p* < 0.0001). We hypothesize that these symptoms may be related to the activity of the SARS-CoV-2 on the ocular surface epithelial cells and glands, causing a microenvironment alteration and consequent instability of the tear film, in particular in patients with pre-existent ocular surface alterations. A similar finding was observed in the oral mucosae, in patients reporting oral symptoms such as dry mouth and amblygeustia, due to damage to the salivary gland caused by the SARS-CoV-2^26^. The SPEED questionnaire used to evaluate the symptomatology of patients was validated in comparison with the most diffuse Ocular Surface Disease Index (OSDI) questionnaire. In fact, the SPEED questionnaire was able to discriminate between asymptomatic and symptomatic subjects related to dry eye disease^27^. However, the mean total score of the SPEED questionnaire in our group was not particularly high, demonstrating mild ocular symptomatology in the evaluated patients.

The Schirmer I test without anesthesia is a standardized test offering an assessment of stimulated reflex.

tear flow, particularly recommended to confirm severe aqueous deficiency dry eye disease^28^. In our group, the mean value for the Schirmer I test did not demonstrate a reduction in tear volume. Nevertheless, we were able to collect a sufficient tear volume to perform RT-qPCR analysis.

Previous reports have documented ocular involvement in patients with confirmed COVID-19, in particular unilateral or bilateral follicular kerato-conjunctivitis with moderate hyperemia and conjunctival injection, corneal sub-epithelial infiltrates, mild eyelid edema, clear watery discharge, photophobia, burning eye, and foreign body sensation^8,9^. Furthermore, pseudomembranous and hemorrhagic conjunctivitis was reported in a patient with severe COVID-19 after the beginning of symptoms. No patients have described a loss of vision, and all cases have reported that the conjunctivitis was resolved in few days. In rare cases, ocular manifestations may represent the first clinical presentation of COVID-19^8,9^. In our group, no patients reported ocular conjunctivitis as the first clinical manifestation of COVID-19. In a large Chinese cohort study on 1099 patients, the presence of “conjunctival congestion” was found in 0.8% of the patients^7^. Although kerato-conjunctivitis is not a common manifestation, other studies documented the onset of ocular symptoms and manifestation, such as conjunctival hyperemia, chemosis and epiphora in 27–31% of patients with COVID-19^10,11^. Zhou et al. documented ocular findings in eight patients of a group of 121 with confirmed COVID-19, reporting an interesting correlation between ocular involvement and the severity of COVID-19^12^. In accordance with previous reports, we noted the occurrence of ocular symptoms in 10 patients (34.5%), conjunctival hyperemia and or chemosis in seven patients (24.1%), and blepharitis findings in eleven patients (37.9%); furthermore, conjunctival findings were significantly correlated with severity of the disease (*p* = 0.002), but not with the duration of the disease, while blepharitis findings were significantly correlated with duration of the disease (*p* = 0.003). Previous studies observed that conjunctival hyperemia and chemosis most commonly occurred in patients with severe systemic disease^11,12^. Several factors may explain the correlation between conjunctival involvement and severity of COVID-19 such as the viral load, and the conjunctival immune response^23–25^; however its real pathogenesis is still not clear. Moreover, conjunctival involvement may be part of the nonspecific, systemic manifestations of diseases like renal failure, cardiopulmonary failure, and carbon dioxide retention, rather than infectious conjunctivitis of COVID-19^13^. Wu et al. showed that white blood cell, neutrophil counts, procalcitonin, C-reactive protein and lactate dehydrogenase were higher in patients who presented with conjunctival involvement when compared to those without any ocular findings^11^. On the other hand, it is well documented that ocular surface disease affects up to 60% of patients admitted to the intensive care unit, due to exposure keratopathy, reduced blink reflex and drying effects of high flow oxygen^29^. Some authors have documented the presence of the virus on the ocular surface in patients with conjunctivitis. Colavita et al. detected viral RNA on the ocular surface in a patient with conjunctivitis and a confirmed diagnosis of COVID-19. The conjunctival swab remained positive for up to 21 days, with a progressive decrease in viral concentration^14^. In two patients with conjunctivitis and confirmed CODIV-19, Zhang et al. documented the presence of SARS-CoV-2 RNA on ocular surface in only one patient^15^. Moreover, Xia et al. reported that SARS-CoV-2 was found on the ocular surface in only one patient with conjunctivitis, and speculated that the tear and conjunctival secretions of patients without conjunctivitis were not an infectious source of SARS-CoV-2^5^. However, the presence of SARS-CoV-2 on the ocular surface has also been found in patients without conjunctivitis; in two patients without ocular involvement and in one with conjunctivitis and critical COVID-19, Zhou et al. reported SARS-CoV-2 positivity in the conjunctival swabs^12^. Furthermore, Xie et al. also demonstrated the presence of SARS-CoV-2 in patients without ocular involvement^6^. These studies demonstrated that the SARS-CoV-2 RNA could be detected from the normal ocular surface of COVID-19 patients, which suggests that the virus is spread through conjunctival contact, and the kerato-conjunctivitis could occur in a predisposed eye and/or when the viral load on the ocular surface is particularly high^6,12^. In the present study, tear analysis did not document the presence of SARS-CoV-2; however, several factors may clarify the negativity of this test. Firstly, tear collection during the initial stages of infection demonstrates an increased probability of SARS-CoV positivity^30^. Only five of the evaluated patients (17.2%) were in the early stage of the disease. Furthermore, the Schirmer I test strips can collect an appropriate volume of tears, but these may contain few epithelial cells and secretions, reducing the probability of identifying the virus. The main limitations of the present study are the small sample size of patients and the different severities and durations of disease at the ophthalmologic assessment and tear collection. Moreover, we did not compare the SPEED questionnaire results with previous results taken prior to the onset of the disease. In conclusion, our data demonstrate the occurrence of different ocular manifestations in patients with COVID-19 but a low probability of detecting the virus in tears and on the ocular surface, when using Schirmer strips as collecting method, especially in patients with a long duration of the disease. Furthermore, it should be noted that ocular surface signs, other than conjunctivitis, can be present in patients affected by COVID-19.

## Methods

This observational study was conducted in 29 hospitalized patients who were admitted to the COVID center at Policlinic Hospital of the University of Messina, Messina, Italy between the 12^th^ April and 14^th^ of May 2020. Informed consent was obtained from each patient. The research was carried out in accordance with the tenets of the Declaration of Helsinki and was approved by the Institutional Review Board of the University of Messina (protocol number 36/20).

All patients tested positive for SARS-CoV-2 according to the WHO interim guidelines^31^. A confirmed case was defined as a positive RT-qPCR result in samples from nasopharyngeal and pharyngeal swabs.

The clinical data of patients, including, age, sex, anamnesis, signs, symptoms, recent exposure history, laboratory findings, and chest computed tomography (CT) scans, were obtained from electronic medical records. The severity of COVID-19 was established according to the recent guidelines^31^.

All patients underwent an ophthalmologic assessment comprising the SPEED questionnaire regarding the frequency and severity of dryness and/or foreign body sensation, ocular pain, burning, and eye fatigue. Each symptom was evaluated as follows: for frequency, from 0 = never to 3 = constant; and for severity, from 0 = absence to 4 = intolerable. The composite score of the questionnaire was obtained by summing up the scores of the frequency and severity parts of the questionnaire; the total score ranged from 0 to 28^32^.

All patients were questioned about previous ocular surgeries, eye disease, use of eye drops, and use of distance spectacles for refractive errors such as myopia, hyperopia, and astigmatism. The anterior segment and the ocular surface of both eyes were examined by an experienced ophthalmologist (AM) using a portable slit lamp (HEINE HSL 150, HEINE Optotechnik, Herrsching, Germany). Conjunctival hyperemia was classified according to the Efron Grading Scales for Contact Lens Complications^33^.

The diagnostic criteria considered for blepharitis comprised the presence of symptoms such as burning, tearing, itching, irritation, photophobia, and findings like hyperemia and/or telangiectasia of eyelid margin, abnormal deposits at the base of the eyelashes, abnormalities of meibomian orifices such as capping, pouting, and obliteration^34^.

Body temperature was measured immediately before the ophthalmologic assessment with a thermo-scan thermometer.

For the Schirmer I test, filter paper strips (Test di Schirmer, Alfa Intes, Casoria, Italy) were applied at the junction between the outer and middle third of the lower lid in both eyes. The moisturized length of the strip was measured after five minutes^35^; afterwards the strips were stored in vials containing universal viral transport media and delivered to the diagnostic laboratory and tested for the presence of SARS-CoV-2. The nasopharyngeal swab was performed on the same day in all patients. Ribonucleic Acid (RNA) extraction was carried out using the NucliSens EasyMag system (bioMérieux, Marcy l’Etoile, France). From a 200-μL tear sample, an aliquot of 100 μL was used for RT-qPCR (Allplex 2019-n CoV Assay Seegene) to identify a target gene specific for SARS-CoV-2 (E gene, RdRP gene, N gen). Data analysis was performed using CFX96, Bio-Rad. An internal control was employed to verify the quality of the extraction in all samples.

### Statistical analysis

Categorical data are described as the frequency rate and percentage, while continuous variables are reported as the mean and standard deviation (SD). Fisher’s Exact test was used to compare categorical variables, and Spearman’s rank correlation analysis was used to evaluate the correlation of ocular symptoms and findings with duration and severity of disease. A *p*-value < 0.05 was considered statistically significant. Statistical analyses were performed using the SPSS 22.0 for Windows package.
